# Unconsciousness as the main nonfocal symptom of anterior circulation transient ischemic attack: A case report

**DOI:** 10.1097/MD.0000000000037343

**Published:** 2024-03-08

**Authors:** Xiaomeng Dong, Ziliang Zhang, Bin Li, Wenbin Ma, Jinbo Chen, Yipeng Su

**Affiliations:** a Department of Neurology, Binzhou Medical University Hospital, Binzhou, China.

**Keywords:** anterior circulation artery, hypoperfusion, ischemic stroke, TIA, unconsciousness

## Abstract

**Rationale:**

Unconsciousness is a nonfocal symptom of transient ischemic attack (TIA) that is frequently observed in patients with vertebrobasilar artery stenosis or occlusion. Conversely, loss of consciousness due to anterior circulation involvement (e.g., middle cerebral artery [MCA]) is a rare occurrence in TIA.

**Patient concerns:**

This report describes a rare case in a 59-year-old woman who experienced recurrent episodes of altered consciousness because of the occlusion or stenosis of her MCAs.

**Diagnoses:**

The diagnosis of the case was updated from TIA to acute cerebral infarction, finally. Following initial loss of consciousness, cranial magnetic resonance imaging (MRI) did not reveal any evidence of acute cerebral infarction. However, following the second and third episodes of unconsciousness, the MRI revealed multiple new acute cerebral infarcts affecting both the cerebral hemispheres. Further evaluation through digital subtraction angiography disclosed complete occlusion of the left MCA and severe stenosis of the right MCA.

**Interventions:**

Early in her illness, the patient was treated with vasodilators, aspirin and atorvastatin. Finally, 2 stents in her right and left MCAs were placed respectively, followed by treatment with aspirin, clopidogrel, and double-dosed atorvastatin calcium. Meanwhile, the patient focused on avoiding conditions which may lead to dehydration in her daily life routine.

**Outcomes:**

The episodes of unconsciousness of this patient were completely resolved. During the 1-year postoperative follow-up, the patient remained clinically stable without any symptoms of unconsciousness, limb numbness or weakness, or dizziness.

**Lessons:**

These findings suggested that hypoperfusion in the bilateral cerebral hemispheres played a pivotal role in precipitating the patient episodes of unconsciousness. This case underscores the possibility that occlusion or severe stenosis in both MCAs can contribute to recurrent episodes of unconsciousness due to hypoperfusion. Moreover, it emphasizes the association between these episodes of unconsciousness and an increased risk of subsequent ischemic stroke.

## 1. Introduction

Transient ischemic attack (TIA) is a medical emergency associated with a high risk of early subsequent stroke. Approximately 10% to 15% of TIA patients develop stroke within 90 days, with half of these strokes occurring within the first 48 hours.^[1]^ Studies have shown that identifying and treating patients with TIA is an effective means of preventing stroke.^[2,3]^ Although the diagnosis of TIA is based on the sudden onset of focal neurological symptoms, such as hemiparesis/hemiplegia and dysarthria, 12% to 31% of TIA patients also experience nonfocal symptoms, such as decreased consciousness, confusion, nonrotatory dizziness, and unsteadiness.^[4]^ There is no difference in the risk of stroke between patients with and without nonfocal symptoms.^[5]^ However, nonfocal symptoms in TIA patients, such as unconsciousness, are more common in the posterior circulation than in the anterior circulation.^[5]^ Furthermore, Toshiya Ishihara et al reported that TIA patients with nonfocal symptoms were more likely to show lesions in the posterior circulation on diffusion-weighted imaging (DWI) and vascular examination than those without nonfocal symptoms, and vascular lesions in the posterior circulation were independently associated with nonfocal symptoms.^[4]^ Unconsciousness in TIA may be caused by transient ischemia of the bilateral hemisphere or brain stem. Severe stenosis of the carotid artery (CA) and vertebrobasilar artery may lead to transient ischemia of the hemisphere and brain stem due to hypoperfusion.^[6]^ Although unconsciousness in TIA is often confused with syncope, epilepsy, or hypoglycemic reactions, research on the pathogenesis, clinical features, and treatment response of unconsciousness in anterior circulation TIA is limited.

This case report aims to highlight this rare nonfocal symptom specifically associated with severe stenosis or occlusion of bilateral middle cerebral arteries (MCAs) and leading to recurrent unconsciousness and acute cerebral infarction.

## 2. Case presentation

**Figure 1.**
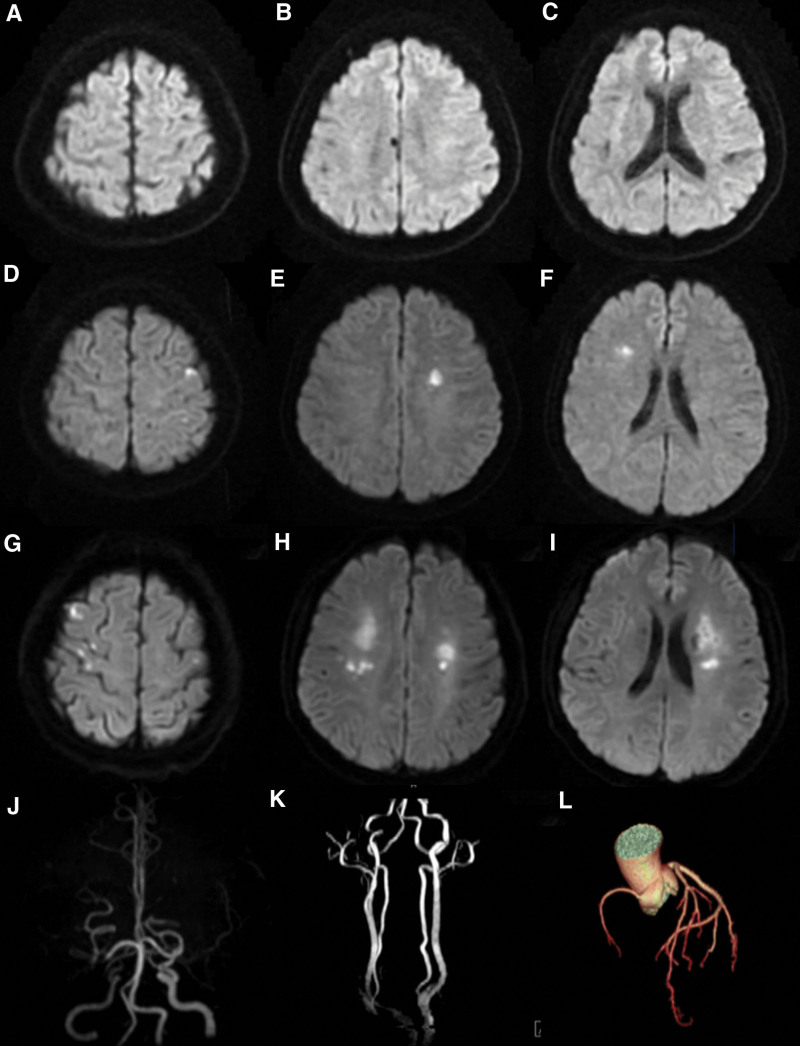
The first brain magnetic resonance imaging shows no acute cerebral infarction on diffusion-weighted imaging (A, B and C), whereas magnetic resonance angiography shows occlusion of the bilateral middle cerebral arteries (J) but no occlusion of the carotid arteries (K). After the second episode multiple acute cerebral infarctions appeared in both hemispheres (D, E and F), whereas several new lesions appeared after the third episode (G,H and I). Coronary angiography showed normal results (L).

**Figure 2.**
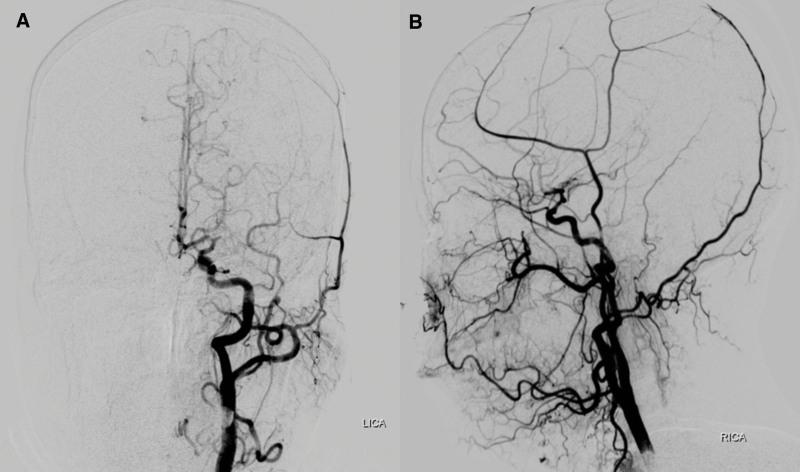
Digital subtraction angiography reveals the occlusion of the left middle cerebral artery (A) and severe stenosis of the right middle cerebral artery and anterior cerebral artery (B).

A 59-year-old woman with hyperlipidemia experienced an episode of unconsciousness while recumbent before seeking medical attention at our hospital. This episode lasted approximately 10 minutes, occurring 30 minutes after she woke up in the morning and before consuming water or breakfast. No seizures were reported by her relatives. She had no history of smoking, alcoholism, or cardiovascular or cerebrovascular diseases and no indications of vasovagal syncope or epilepsy. Physical examination revealed stable vital signs, with no neurological or cardiorespiratory abnormalities. The initial brain magnetic resonance imaging (MRI) did not show any acute cerebral infarction on DWI (Fig. 1A~C), whereas magnetic resonance angiography showed the occlusion of the bilateral MCAs (Fig. 1J). Transcranial Doppler ultrasound (TCD) examination detected slow blood flow and spectral disturbance in the bilateral MCAs. No cardiac abnormalities were found on Holter monitoring, transthoracic echocardiography, or coronary angiography (Fig. 1L), and the TCD foaming test did not identify a right-to-left shunt (Fig. 2C). Video electroencephalogram did not detect epileptic waves during the onset or interval of unconsciousness. A comprehensive battery of laboratory tests, including those for complete blood count, biochemistry, coagulation markers, immune markers (e.g., anti-O and rheumatoid factor), anti-nuclear extract antibody, anti-cardiolipin antibody, lupus anticoagulant, antineutrophilic cytoplasmic antibody, hepatitis B, human immunodeficiency virus, and syphilis, were performed. The results showed abnormal elevations only in the blood cholesterol and low-density lipoprotein levels, indicating atherosclerosis as the primary cause of severe cerebral artery stenosis. On admission, the patient was injected with Danshen, a traditional Chinese medicine for vasodilation, and took aspirin and atorvastatin.

Two days later, the patient experienced a second episode of unconsciousness in the hospital shortly after waking up and before consuming breakfast or water. This episode presented with additional clinical manifestations, including pale complexion, profuse sweating, and urinary incontinence. Following this episode, an electrocardiogram revealed a drop in the blood pressure from 110/70 mm Hg to 82/52 mm Hg and a decrease in the heart rate from 78 beats/min to 48 beats/min. Furthermore, the peripheral blood sugar was 5.5 mmol/L, ruling out hypoglycemia as the cause of loss of consciousness (LOC). The second episode of unconsciousness lasted approximately 15 minutes, and on regaining consciousness, the patient experienced focal neurological deficits that lasted for almost 30 minutes, characterized by numbness and weakness in the left limb. No further abnormal symptoms occurred. The electrocardiogram findings were normal, without any arrhythmias such as atrial fibrillation or severe atrioventricular block. The physical examination performed 15 minutes after the second episode revealed a blood pressure of 110/62 mm Hg in the recumbent position and 106/60 mm Hg in the upright position. The second MRI examination conducted after this episode revealed multiple acute cerebral infarctions in both hemispheres (Fig. 1D~F).

Approximately 13 days later, the patient experienced another episode characterized by approximately 30 minutes of continuous confusion, followed by numbness and weakness in the right limb and aphasia for almost 30 minutes after waking up. Compared to the second MRI, the third MRI showed new acute cerebral infarction lesions in both the cerebral hemispheres (Fig. 1G~I). Digital subtraction angiography confirmed the occlusion of the left MCA (Fig. 2A) and severe stenosis of the right MCA and anterior cerebral artery (ACA) (Fig. 2B). Collateral compensation through the posterior cerebral artery was relatively well established on the right side and through the ACA on the left side.

## 3. Outcome and follow-up

The patient underwent stent placement in both the left and right MCAs, followed by treatment with aspirin, clopidogrel, and double-dosed atorvastatin calcium. In her daily life routine, the patient focused on increasing her food and water intake while taking precautions to avoid conditions leading to dehydration, such as vomiting and diarrhea. The patient postoperative course was uneventful, and the episodes of unconsciousness were completely resolved. During the 1-year postoperative follow-up, the patient remained clinically stable without any symptoms of unconsciousness, limb numbness or weakness, or dizziness.

## 4. Discussion and conclusions

Since the highest risk for stroke is in the first 48 hours following TIA symptom onset, it is critical to identify the mechanisms of TIA and initiate appropriate treatment at the earliest.^[7]^ Notably, almost one-third of TIA patients also experience nonfocal symptoms, including reduced consciousness, confusion, nonrotatory dizziness, and unsteadiness; however, TIA diagnosis primarily hinges on the sudden onset of focal neurological symptoms, such as aphasia, dysarthria, hemiparesis/hemiplegia, and facial paralysis.^[4,5]^ Moreover, non-rotatory dizziness, paresthesia, and amnesia are the most commonly seen nonfocal symptoms of TIA.^[7]^ Transient LOC (TLOC) is defined as a state of real or apparent LOC with loss of awareness, characterized by amnesia for the period of unconsciousness, abnormal motor control, loss of responsiveness, and a short duration.^[8]^ In cases of insufficient cerebral blood flow (CBF), the brain can maintain its function only for a few seconds.^[6]^ A sudden cessation of CBF for as less as 6 to 8 seconds can cause complete LOC.^[9]^ The causes of TLOC vary widely and may encompass syncope, metabolic disorders, epileptic seizures, psychogenic disorders, and even TIA itself.^[10]^ TIA of the vertebrobasilar system can cause LOC, but it is always accompanied by local symptoms, including limb weakness, gait and limb ataxia, vertigo, diplopia, nystagmus, dysarthria, and oropharyngeal dysfunction. Less than 1% of patients with vertebrobasilar ischemia show only one presenting symptom.^[11]^

Notably, unconsciousness as a nonfocal symptom of TIA is more frequent in cases of posterior circulation occlusion and less frequent in anterior circulation occlusion (e.g., internal carotid artery or MCA), and it is often associated with poor outcomes.^[7]^ This association between nonfocal symptoms and the posterior circulation could be because some nonfocal symptoms defined in previous studies may be manifestations of ischemia in the posterior circulation. A single-center study focusing on posterior circulation found that symptoms such as dizziness, nausea, vomiting, LOC, bilateral limb weakness, and hearing loss were frequently observed in patients with ischemia in the vertebrobasilar artery territory.^[4]^ TIA related to a CA does not usually cause TLOC, except in orthostatic TIA, wherein a combination of multiple stenoses of cerebral arteries and orthostatic hypotension is observed. This may rarely result in repetitive, orthostatic, short-lasting, and stereotyped TIAs.^[12,13]^ Cases of brief LOC due to occlusion of bilateral CAs or MCAs have few been reported.

**Figure 3.**
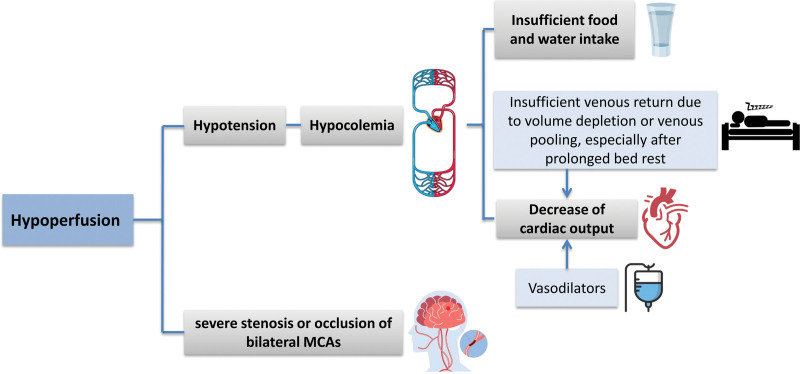
The mechanisms potentially lead to episodes of unconsciousness in this case. Other underlying factors cannot be ruled out.

We propose possible mechanisms to explain the puzzling episodes of unconsciousness of our patient as follows and provide a comprehensive summary in Figure 3. Our patient had severe stenosis or occlusion of bilateral MCAs, which resulted in multiple episodes of unconsciousness accompanied by hemiplegia and aphasia. Physical examination indicated that hyperlipidemia was the primary cause of the cerebral atherosclerosis in this patient. The presence of stenosis in the bilateral MCAs necessitates the maintenance of a high systolic pressure to ensure adequate brain perfusion. This condition makes the patient more susceptible to hypoperfusion during episodes of hypotension. Notably, the patient experienced unconsciousness, particularly in the morning when recumbent and on an empty stomach, despite having relatively low blood pressure. Low blood volume resulted in reduced blood pressure, which in conjunction with cerebral artery stenosis collectively contributed to these episodes of unconsciousness. Furthermore, decreased venous return may result in diminished cardiac output, subsequently causing hypotension.^[8]^ Extensive auxiliary examinations, including cardiac Holter monitoring, transthoracic echocardiography, coronary angiography, and TCD foaming test, revealed no structural abnormalities in the patient heart and great vessels nor did they indicate any arrhythmias explaining the decreased cardiac output. We postulated that insufficient venous return due to volume depletion or venous pooling, especially after prolonged bed rest, reduced the cardiac output, ultimately triggering the episodes of unconsciousness, particularly when the patient was recumbent in the morning upon waking up.

Generally, unconsciousness due to TIA is uncommon in cases of unilateral CA or MCA stenosis because at least one cerebral hemisphere is adequately perfused. In our patient, angiography confirmed left MCA occlusion with well-established ACA collateral compensation, as well as severe right MCA and ACA stenoses with well-established posterior cerebral artery collateral compensation. Unconsciousness might occur when hypotension causes decreased blood supply to these collateral compensatory routes. Recurrent unconsciousness in this patient was attributed to the hypoperfusion of both hemispheres, a condition exacerbated by the use of vasodilators and fluid loss due to insufficient food and water intake.

Syncope, defined as TLOC due to cerebral hypoperfusion, is characterized by its rapid onset, short duration, and spontaneous complete recovery.^[8,14]^ It can be easily misdiagnosed as TIA, especially when accompanied by unconsciousness. A well-known statement by Van Dijk highlights this distinction: “In clinical practice, TIA is characterized by focal neurological signs without TLOC, whereas syncope is characterized by TLOC without focal neurological signs.”^[15]^ The 2018 European Society of Cardiology guidelines^[8]^ categorize syncope into the following 3 primary types: reflex or neurologically mediated syncope related to a specific trigger; syncope due to orthostatic hypotension, defined by a drop of > 20 mm Hg in the systolic blood pressure or > 10 mm Hg in the diastolic blood pressure after standing for 3 minutes; and cardiac syncope caused by structural diseases of the heart and great vessels. Although there are several types of syncope, they all culminate in the occurrence of global cerebral hypoperfusion, which results from the inability of the circulatory system to maintain adequate blood pressure for efficient CBF.^[16]^

**Figure 4.**
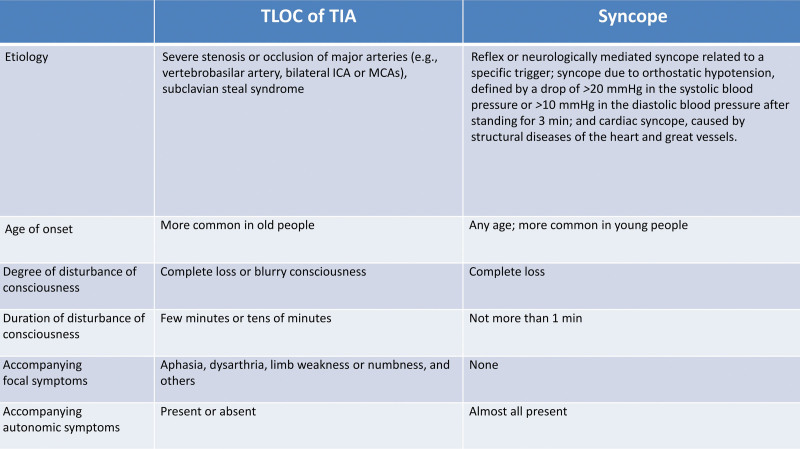
Differences between characteristics of transient loss of consciousness in transient ischemic attack and syncope. ICA = internal carotid artery, MCA = middle cerebral artery, TIA = transient ischemic attack, TLOC = transient loss of consciousness.

While there are shared characteristics in the presentation of TLOC between syncope and TIA, including rapid onset, short duration, and complete and spontaneous recovery, they differ significantly in terms of etiology, age of onset, degree and duration of LOC, and accompanying symptoms (Fig. 4). In this patient, the unconsciousness ranged from complete LOC to a state of blurred consciousness, with each episode lasting 10 to 30 minutes. Following the recovery of consciousness, the patient exhibited multiple focal nervous system deficits, such as aphasia and unilateral limb numbness and weakness, along with autonomic nervous symptoms, such as sweating and pallor. Cardiac syncope, orthostatic hypotensive syncope, and epilepsy were ruled out through a battery of auxiliary examinations, including Holter monitoring, transthoracic echocardiography, coronary angiography, TCD foaming test, supine blood pressure measurement, and video electroencephalogram. Peripheral blood glucose levels were monitored during the episodes of unconsciousness, and hypoglycemic reactions were ruled out.

Although there is ongoing debate regarding the role of stents in the treatment of MCA stenosis, it remains crucial in cases of severe stenosis or occlusion of bilateral MCAs that result in severe clinical symptoms due to hypoperfusion. In this patient, 2 stents were successfully placed in the left and right MCA, respectively, and administration of medications, including aspirin, clopidogrel, and double-dosed atorvastatin calcium, was continued after the procedure. Correcting hypotension and hypovolemia is paramount to maintaining systemic perfusion levels necessary for organ function. Adequate hydration and salt intake are recommended to ensure sufficient blood volume. Patients should also be advised to avoid vasodilators, alcohol, low fluid intake, triggers for diarrhea or vomiting, and other factors that can lead to reduced blood volume.^[8]^ Following surgical treatment, medication management, and lifestyle changes such as increased fluid and food intake, our patient showed a favorable prognosis. No recurrent symptoms of unconsciousness and limb numbness or weakness were observed even after the 1-year follow-up period.

DWI MRI is more sensitive than computed tomography for detecting acute ischemia, and it has been shown that almost one-third of patients initially diagnosed with TIA show infarcts on DWI MRI.^[17]^ A previous study reported that the risk of stroke was similar in patients with or without nonfocal symptoms.^[5]^ Our patient exhibited new cerebral infarction lesions on DWI following the second and third TIA episodes, wherein nonfocal symptoms, including unconsciousness, were observed along with some focal symptoms. Consequently, the diagnosis was updated from TIA to acute cerebral infarction, aligning with the new “tissue-based” definition of TIA.^[17]^ This underscores the importance of recognizing nonfocal TIA symptoms such as LOC and initiating prompt treatment to reduce the risk of stroke following TIA.

In conclusion, this case report underscores the significance of differential diagnoses for recurrent episodes of unconsciousness. It highlights the importance of recognizing nonfocal symptoms associated with anterior circulation TIA and emphasizes the necessity of addressing underlying severe stenosis of the MCA in patients experiencing recurrent loss of consciousness.

## Acknowledgments

The authors would like to thank the patient for consenting to the publication of this case and Bullet Edits Limited for the linguistic editing and proofreading of the manuscript.

## Author contributions

**Data curation:** Bin Li, Wenbin Ma, Yipeng Su.

**Formal analysis:** Jinbo Chen.

**Funding acquisition:** Jinbo Chen.

**Writing – original draft:** Xiaomeng Dong.

**Writing – review & editing:** Xiaomeng Dong, Ziliang Zhang, Yipeng Su.
